# A Novel High Throughput Biochemical Assay to Evaluate the HuR Protein-RNA Complex Formation

**DOI:** 10.1371/journal.pone.0072426

**Published:** 2013-08-12

**Authors:** Vito G. D’Agostino, Valentina Adami, Alessandro Provenzani

**Affiliations:** 1 Laboratory of Genomic Screening, Centre for Integrative Biology, University of Trento, Mattarello, Trento, Italy; 2 High Throughput Screening core facility, Centre for Integrative Biology, University of Trento, Mattarello, Trento, Italy; Universität Stuttgart, Germany

## Abstract

The RNA binding protein HuR/ELAVL1 binds to AU-rich elements (AREs) promoting the stabilization and translation of a number of mRNAs into the cytoplasm, dictating their fate. We applied the AlphaScreen technology using purified human HuR protein, expressed in a mammalian cell-based system, to characterize *in vitro* its binding performance towards a ssRNA probe whose sequence corresponds to the are present in TNFα 3’ untranslated region. We optimized the method to titrate ligands and analyzed the kinetic in saturation binding and time course experiments, including competition assays. The method revealed to be a successful tool for determination of HuR binding kinetic parameters in the nanomolar range, with calculated *Kd* of 2.5±0.60 nM, *k*_*on*_ of 2.76±0.56*10^6^ M^-1^ min^-1^, and *k*_*off*_ of 0.007±0.005 min^-1^. We also tested the HuR-RNA complex formation by fluorescent probe-based RNA-EMSA. Moreover, in a 384-well plate format we obtained a Z-factor of 0.84 and an averaged coefficient of variation between controls of 8%, indicating that this biochemical assay fulfills criteria of robustness for a targeted screening approach. After a screening with 2000 small molecules and secondary verification with RNA-EMSA we identified mitoxantrone as an interfering compound with rHuR and TNFα probe complex formation. Notably, this tool has a large versatility and could be applied to other RNA Binding Proteins recognizing different RNA, DNA, or protein species. In addition, it opens new perspectives in the identification of small-molecule modulators of RNA binding proteins activity.

## Introduction

The stability of a specific mRNA is dependent upon both cis-elements and trans-acting factors such as RNA binding proteins (RBPs). HuR/ELAVL1, initially discovered to be essential for the development of the *Drosophila melanogaster* nervous system [1], is a widely studied RBP that binds preferentially to AU-rich elements (AREs) mainly localized in the 3’ untranslated region (UTR) of mRNAs [2,3], although other consensus binding elements have emerged [4], mainly with a stabilizing effect in the target mRNA. HuR shares with closely related RBPs of the Embryonic Lethal Abnormal Vision (ELAV) protein family a common characteristic structure of three highly conserved RNA recognition motifs (RRMs), of which the two tandem N-terminal RRM domains confer high affinity for ARE sequences [5,6]. The functional relevance of HuR-driven post-transcriptional regulation is pivotal in many pathologies, wherein occurrence and progression tightly correlate with a dysregulation in mRNA stability, including chronic inflammation, cardiovascular diseases, cancer, and also resistance to chemotherapy [7–11]. High turnover mRNAs that form complexes with HuR (see review [7]) are usually studied by ribonucleo-immunoprecipitation coupled to immunoblotting/RT-PCR or by RNA-Electrophoresis Mobility Shift Assays (REMSAs). However, these approaches have several limitations due to the necessity of good antibodies for immunoprecipitation and to the assumption that complexes observed in non-denaturing gels are a good approximation of the cellular events. We therefore decided to develop a biochemical tool, based on AlphaScreen technology, that could complement traditional biochemical methods in the rapid and sensitive evaluation of HuR-RNA interaction and of competition with other trans-acting factors (direct or indirect protein–protein interactions). To this aim, we exploited the affinity between HuR and the AU-rich region of the TNFα 3’ UTR mRNA for the development of our tool. Indeed, several reports have described the stabilization function and translational impact of HuR towards the TNFα mRNA [12–15]. Here we show that full-length human HuR protein can be functionally expressed in mammalian cells and the binding kinetic parameters, characterizing the complex formation with a RNA probe whose sequence corresponds to the AU-rich region of the TNFα 3’ UTR mRNA, can be quantified in the nanomolar range. Furthermore, we show that this tool can substitute standard REMSAs for quantitative evaluation of the protein-RNA association and the feasibility of the AlphaScreen assay for high throughput screening (HTS) applications.

## Material and Methods

### Preparation and detection of recombinant HuR and TTP proteins

Full-length human HuR/ELAVL1 cDNA (NM001419) sequence was amplified from MCF7-cells retro-transcribed RNA and inserted into the pCMV6-AC-Myc-His PrecisionShuttle vector (Origene Technologies; PS100006) by using the forward (5’-GCC GCGATCGC CATGTCTAATGGTTATGA-3’) and reverse (5’-CGT ACGCGT TTTGTGGGACTTGTTGG-3’) primers containing the SgfI and the MluI restriction sites, respectively. Frame and sequence of the full-length ORF, with the Myc-His tag-encoding sequence located at the 3’-end, was confirmed by sequencing. The recombinant vector pCMV6-HuR was transfected (3 µg per 80% cell-confluent 10 cm dish) in HEK293T (ICLC; HTL04001) cells by using Lipofectamine^®^ 2000 transfection reagent (Life Technologies; 11668-019) according to the manufacturer’s protocol. HEK293T were grown in complete medium (10% DMEM Lonza BE12-917F, 10% FBS Lonza 14-503F, 2 mM Glutamine Lonza 17-605, 100 units/ml penicillin plus 100 µg/ml streptomycin Lonza DE17-605E) in a humidified 5% CO_2_ atmosphere at 37° C. Cells were harvested 48 hr post transfection and sonicated (90 Hz of amplitude for 3 cycles of 15 sec, paused by 1 min) at 4° C in buffer W (20 mM NaH_2_PO_4_, 0.5 M NaCl, 20 mM imidazole, pH 7.4) supplemented with Protease Inhibitor Cocktail (Sigma-Aldrich; P8340). To induce rHuR phosphorylation, transfected cells were treated with cyclosporine A (CsA) for 3 hr at final concentration of 4 µM. Recombinant HuR-Myc-His (rHuR) protein was purified by affinity chromatography with HisTrap HP resin (GE Healthcare; 17-5248-01) and eluted with imidazole gradient ranging from 62.5 to 500 mM. rHuR was dialyzed by Zeba^TM^ Spin Desalting Columns (Thermo Scientific; 89890), following the recommended protocol, to remove imidazole residues and it was stored at -80° C in buffer S (20 mM NaH_2_PO_4_, 100 mM NaCl, 50 mM glycine, 10% glycerol, pH 7.5). pcDNA3 vector expressing mouse (NM_011756.4) recombinant His-Strep-TTP (rTTP) protein was a kind gift from Prof. Gaestel (Hannover Medical School, Germany). HEK293T were transfected with 2 µg of plasmid, per 10 cm dish, and were lysed 24 hr later. rTTP was purified using streptactin resin (IBA TAGnologies; 2-1202-001) as described previously [16] and eluted in buffer W containing 1 mM desthiobiotin (IBA TAGnologies; 2-1000-002). Zeba^TM^ Spin Desalting Columns were used for salts decontamination as described above and protein was stored at -80° C in buffer S. Recovered rHuR and rTTP proteins were analyzed by Coomassie staining on 15%-SDS-PAGE and relative protein concentration was determined for each preparation using bovine serum albumin (BSA) standards and densitometric quantification (ImageJ 1.4 software, NIH) of corresponding bands on acrylamide gels. Western blot analysis were performed on rHuR using a mouse anti-HuR antibody (Santa Cruz Biotech; sc-5261) as described previously [8]. Phosphorylated rHuR protein was detected using a rabbit anti-phosphoserine antibody (Merck Millipore; ab1603).

### AlphaScreen assay

Amplified Luminescent Proximity Homogenous Assay (Alpha) was applied to study the interaction between rHuR and the specific ARE-bearing sequence present in 3’ UTR of TNFα mRNA. Biotinylated single-stranded Bi-TNF (5’-Bi-AUUAUUUAUUAUUUAUUUAUUAUUUA-3’), Bi-TNFneg (5’-Bi-ACCACCCACCACCCACCCACCACCCA-3’), Cy-TNF (5’-Cy3-AUUAUUUAUUAUUUAUUUAUUAUUUA-3’), untagged U-TNF (5’-AUUAUUUAUUAUUUAUUUAUUAUUUA-3’) TNFα and Bi-COX (5’-Bi-UAUUAAUUUAAU UAUUUAAUAAUAUUU-3’) COX-2 RNA probes were purchased from Eurofins MWG Operon. The assays were performed in 384-well white OptiPlates (PerkinElmer; 6007299) in a final volume of 25 µl and optimized by titrating both interacting partners (in order to determine the optimal protein: RNA ligand ratio). Values out of the “hooking zone”, where quenching of the signal is due to an excess of the binding partner, were determined for the optimal concentrations of probe and protein [17]. All reagents were tested in the nanomolar range using the AlphaScreen c-Myc detection kit (PerkinElmer; 6760611M) and reacted in buffer A (25 mM HEPES pH 7.4, 100 mM NaCl, 0.1% BSA). For the optimization of the assay, a series of concentration of rHuR (0–3 nM) was incubated with different concentration of Bi-TNF probe (0–100 nM) in matrix configuration. Subsequently, anti-c-Myc-Acceptor beads (PerkinElmer) (20 µg/ml final concentration) were added and the reaction was placed in the dark at room temperature for 30 min. Then streptavidin-Donor beads (20 µg/ml final concentration) were added and reaction was incubated at room temperature for 90 min to reach equilibrium. Fluorescence signals were detected on Enspire plate reader instrument (PerkinElmer; 2300-001A) and specific interactions were quantified by subtracting the signal of the background, calculated in the absence of the protein and/or of the probe and with protein elution buffer only (nonspecific binding).

### Saturation and Time course experiments

Saturation binding experiments were carried out incubating a series of concentration of Bi-TNF and Bi-TNFneg probes (0–100 nM) with rHuR (1 nM) and beads (20 µg/ml) in buffer A, as described above. Assays were performed in quadruplicate with four different protein preparations. Equilibrium dissociation constants (*Kd*) were determined from nonlinear regression fits of the data according to a 1-site binding model in GraphPad Prism®, version 5.0 (GraphPad Software, Inc., San Diego, CA). Fitting values have been reported as averaged mean±standard deviation of all the experiments. In time course experiments, rHuR (1 nM) was preincubated with anti-c-Myc-Acceptor beads (20 µg/ml) for 15 min (complex 1) and Bi-TNF probe, ranging from 3.125 to 50 nM, was preincubated with streptavidin-Donor beads (20 µg/ml) for 15 min (complex 2). Assays were started at different time points by mixing equal volumes of complex 1 and complex 2 and the signals of the whole 384-well plate were detected at the end of the time course. The kinetics was performed in duplicate with two different protein preparations. Association and dissociation rate constants were determined from nonlinear regression fits of the data according to association kinetic model of multiple ligand concentration in GraphPad Prism®, version 5.0. The resulting *Kd* values obtained by *k*_*off*_/*k*_*on*_ ratio were compared with the *Kd* of saturation binding experiments.

### Competitive assays

Unlabeled RNA oligos (U-TNF) were mixed, at different concentration, with Bi-TNF probe (50 nM). These substrates were reacted with 1 nM of rHuR in the experimental condition of saturation binding and the signals were acquired when the reaction reached equilibrium (60 min later). Protein competition assays were carried out by performing rHuR-Bi-TNF binding reaction, with Acceptor and Donor beads, for 15 min and then different nanomolar rTTP and BSA concentrations were added. Nonspecific binding was subtracted and percentage of inhibition were plotted for the assays. The equilibrium dissociation constants (*ki*) of U-TNF and rTTP ligands were determined from nonlinear regression fits of the data according to 1-site fit Ki model in GraphPad Prism®, version 5.0, by keeping constant the concentration (50 nM) and the *Kd* (2.5 nM) of the labeled Bi-TNF probe and by assuming that the binding was reversible and at equilibrium.

### RNA-Electrophoresis Mobility Shift Assay (REMSA)

rHuR and Cy-TNF RNA probe were reacted in low micromolar concentration, as indicated, in buffer E (20 mM HEPES pH 7.5, 50 mM KCl, 0.5 µg BSA, 0.25% Glycerol) in a final volume of 20 µl at room temperature. For supershift experiments 0.5 µg of anti-HuR antibody was added 10 min after preincubation of ligands. The reaction mix was then loaded onto 6% native polyacrylamide gel containing 0.5% Glycerol. Run was performed in 0.5X TBE buffer at 45 V and 4° C for 3 hr. Free and complexed RNA probe were detected with Typhoon Instrument (GE Healthcare; 00-4277-85 AC) using filters for red light emission detection.

### High Throughput Screening of small molecules

AlphaScreen assay was optimized in a final volume of 20 µl, with 1 nM of rHuR, 50 nM of RNA probe, and 20 µg/ml of beads. Experiments for assay quality and robustness evaluation were initially performed using two 384-Optiplates with random distribution of Bi-TNF positive and Bi-TNFneg negative controls. In the primary screening of the small molecules, controls (Bi-TNF positive, Bi-TNFneg negative, DMSO) were located in the first and in the last two columns of the 384-Optiplates and each compound was tested in duplicate, in single dose and in different plates. Molecules were tested at the final concentration of 25 nM and all the dispensation steps were performed by the 96 channels pipetting head of a Tecan EVO 200 (Tecan, Switzerland). Primary screen was performed with a 2000 molecules library (Spectrum Collection, MicroSource Discovery, USA), containing 60% of clinically used drugs, 25% of natural products and 15% of other bioactive components. Data and statistics were analyzed by GraphPad Prism®, version 5.0, software. Z-factor value was calculated with the equation: 1-[3(SD_p_+SD_n_)/|M_p_-M_n_|], while Z-score values were calculated as: (X-M_p_)/SD_p_, where SD is the standard deviation, M is the mean, (p) and (n) are positive and negative controls, respectively, and X indicates fluorescence intensity of compound [18]. Competitive REMSA were performed as described above but using 0.5 µM of compound, 0.2 µM of rHuR protein and 0.5 µM Bi-TNF probe.

## Results

### Purification and functional binding of recombinant HuR proteins

**Figure 1:**
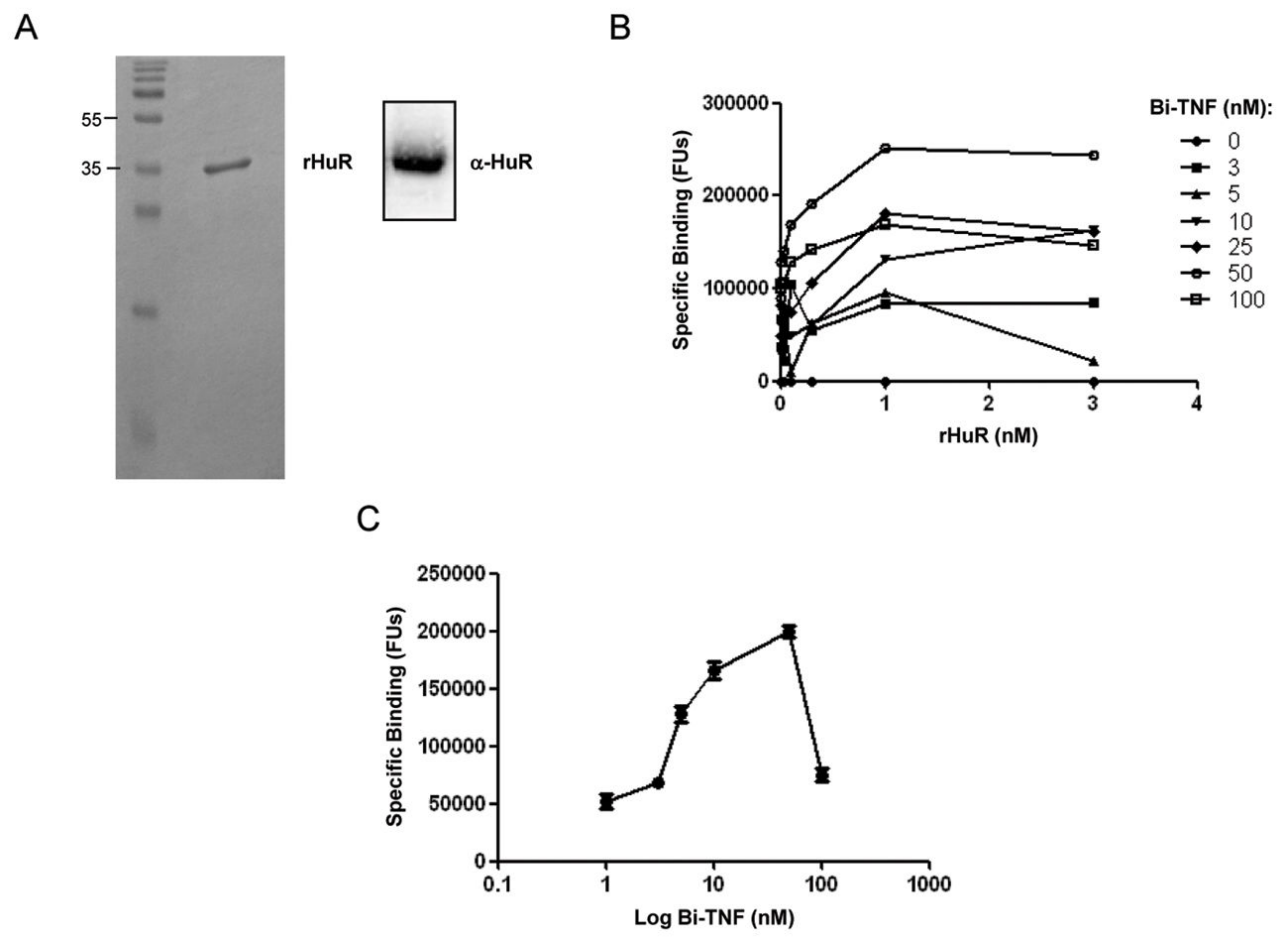
Purification of rHuR and optimization of the AlphaScreen assay. **A**) Representative 15% SDS-PAGE and Coomassie staining of purified rHuR protein (80 ng) recovered after Zeba^TM^ Spin Desalting Columns dialyzation and western blot on the same sample (20 ng) using a polyclonal anti-HuR antibody. **B**) Bi-TNF RNA probe and rHuR protein double titration to determine optimal ligand concentrations with the AlphaScreen anti-c-Myc-Acceptor and streptavidin-Donor beads of the c-Myc detection kit (PerkinElmer), resulting in 1 nM and 50 nM for rHuR and Bi-TNF, respectively. **C**) Bi-TNF titration with 1 nM of rHuR. “Hooking effect” is shown for concentrations over 50 nM of RNA ligand (as the point at 100 nM in the log scale). Mean and standard deviation values derive from four independent experiments with four rHuR protein preparations.

**Figure 2:**
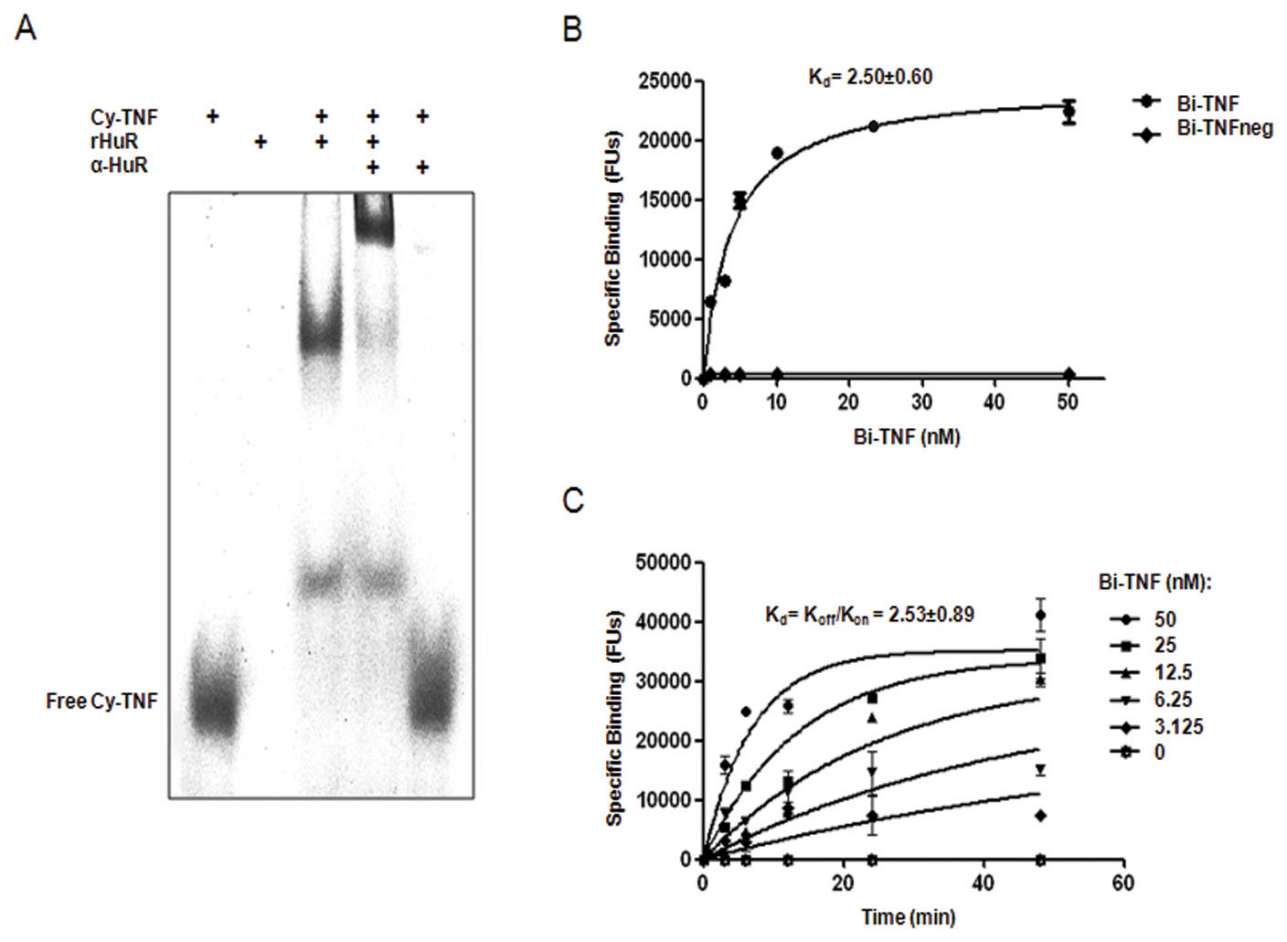
Characterization of the functional binding of rHuR to the AU-rich RNA substrate. **A**) REMSA showing the binding capability of rHuR (0.5 µM) resulting in the presence of an higher molecular weight protein-RNA complex with respect to the free Cy-TNF RNA probe (0.5 µM). The supershift caused by the anti-HuR antibody (1 µg) indicates the presence of at least a ternary complex and the qualitative binding of rHuR. **B**) Saturation binding experiments. Equilibrium dissociation constants (*Kd*) were determined from nonlinear regression fits of the data according to a 1-site binding model in GraphPad Prism®, version 5.0. Mean and standard deviation values derive from four independent experiments with four rHuR protein preparations. **C**) Time course experiments. Association (*K*_*on*_) and dissociation (*K*_*off*_) rate constants were determined from nonlinear regression fits of the data according to association kinetic model of multiple ligand concentration in GraphPad Prism®, version 5.0. Mean and standard deviation values derive from two independent experiments with two rHuR protein preparations.

We produced human recombinant HuR (rHuR) protein from HEK293T cells. By transient transfection of HEK293T cells with pTrueORF-HuR plasmid we purified rHuR protein with both c-Myc and His6X tag in the C-terminal region. rHuR showed high purity after four steps of imidazole elution and western blot analysis confirmed a single band of the expected size (~38 kDa) (Figure **1A**). The average protein yield, per 10 cm-dish with 80% confluent cells at the moment of transfection, was 1.5 µg. In order to characterize the binding activity of rHuR to the TNFα ARE consensus we applied AlphaScreen technology using a 5’-biotinylated ssRNA sequence as substrate [15] (Bi-TNF). We optimized the assay to identify the best molar ratio between the two interacting partners coupled with anti-c-Myc-Acceptor and Streptavidin-Donor beads (Figure **1B**); the optimal concentration was 1 nM and 50 nM for rHuR and Bi-TNF, respectively. The “hooking effect”, where high concentrations of analyte exceed the binding capacity of the beads and lead to a reduced signal, was determined by single titrations showed in Figure **1C**. We identified the formation of rHuR-RNA probe complex using nondenaturing and non cross-linked REMSA (Figure **2A**) after mixing equimolar amount (0.5 µM) of protein and Cy3-tagged TNF probe (Cy-TNF). As shown in the mobility shift assay, rHuR clearly caused the RNA probe electrophoretic retardation detectable as one prominent band, and the addition of the anti-HuR antibody in the binding reaction was able to produce the typical supershift of the RNA-protein complex. In these steps, we produced and purified the human recombinant HuR protein from HEK293T cells in its active form able to bind to ARE probes in two different biochemical assays. In comparison, optimal protein concentration for AlphaScreen assays resulted in 500 times lower than the amount required for a standard REMSA.

### Characterization of rHuR binding to Bi-TNF probe

In accordance with AlphaScreen optimization data, rHuR binding kinetic was dependent on the concentration of the labeled Bi-TNF oligonucleotide and specific binding was consistent with the presence of AU-rich sequences in the substrate. Saturation binding experiments (Figure **2B**) showed that rHuR has high affinity for Bi-TNF, with *Kd* value of 2.5±0.60 nM, which is in line with *Kd* values of other reports characterizing human HuR protein expressed in *E. coli* cells [19,20]. As expected, absence of the Uridine tandem in the Bi-TNFneg probe caused the loss of ligand interactions, as shown by the fluorescence signal near to background, making this RNA probe an ideal negative control. Time course experiments showed that binding of rHuR to Bi-TNF probe was both time and dose dependent (Figure **2C**). Data were globally fitted using the association kinetic model of multiple ligand concentration: derived association (*k*_*on*_ of 2.76±0.56*10^6^ M^-1^ min^-1^) and dissociation (*k*_*off*_ of 0.007±0.005 min^-1^) rates indicated a very high affinity of the rHuR protein towards this RNA substrate and a low dissociation probability, as also reported by Kim et al 2011. According to the law of mass action, the equilibrium binding constant *Kd* calculated as *k*_*off*_ */k*_*on*_ was absolutely indistinguishable from *Kd* values obtained by saturation experiments, indicating that the thermodynamic equilibrium was reached after 20 min of incubation. To assess if AU rich elements derived from different 3’ UTR than TNFα showed similar properties, we chose to examine the binding affinity towards a ssRNA derived from the ARE of the COX-2 3’ UTR [20]. In saturation binding experiments the *Kd* was 4.583±1.2 nM, indicating that this assay has a broad potential to investigate many different mRNA targets (Figure **S1**).

### Utilization in competition experiments

**Figure 3:**
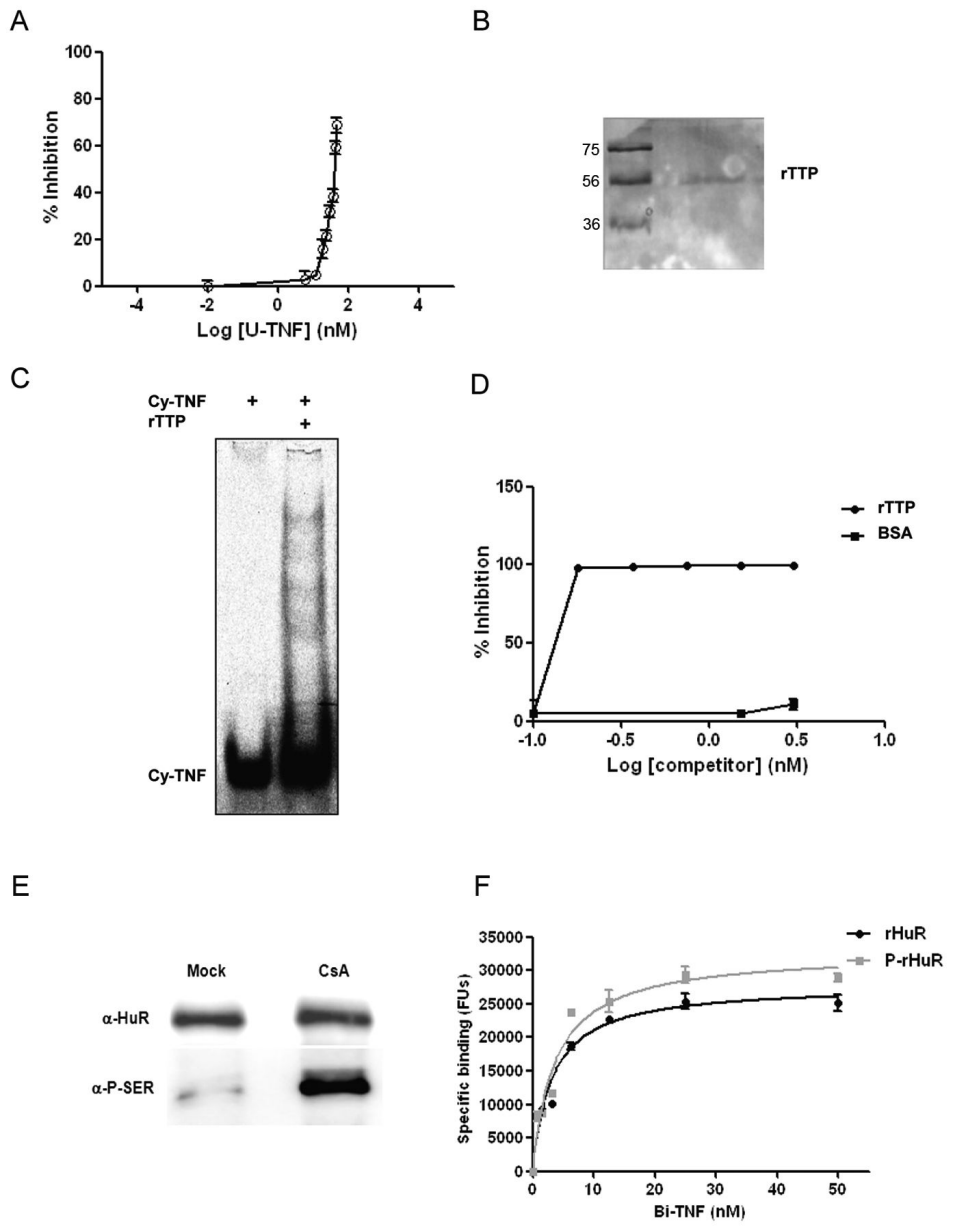
Competition assays with unmarked RNA oligonucleotide and with rTTP protein. **A**) The percentage of inhibition increased as function of untagged TNFα RNA probe (U-TNF) concentration. U-TNF was added to the reaction together with Bi-TNF probe and signals were detected at equilibrium. *Ki* values were determined from nonlinear regression fits of the data according to 1-site fit *Ki* model in GraphPad Prism®, version 5.0, by keeping constant the concentration (50 nM) and the *Kd* (2.5 nM) for the labeled Bi-TNF probe. Mean and standard deviation values derive from two independent experiments. **B**) Coomassie staining of purified and recovered Zeba^TM^ Spin desalted rTTP protein (25 ng) loaded on 15% SDS-PAGE. **C**) REMSA showing rTTP (0.1 µM) complexed with Cy-TNF RNA probe (0.5 µM reacted and loaded on native gel). **D**) Competitive AlphaScreen assay as a function of rTTP concentration. Equal amounts of BSA were independently reacted as negative control. *Ki* values were determined from nonlinear regression fits of the data according to 1-site fit *Ki* model in GraphPad Prism®, version 5.0, by keeping constant the concentration (50 nM) and the *Kd* (2.5 nM) for the labeled Bi-TNF probe. Mean and standard deviation values derive from two independent experiments with two rTTP protein preparations. **E**) Western blot showing rHuR purified from control (Mock; DMSO) and CsA [4 µM] stimulated HEK293T cells. After 3 hr of treatment the total amount of purified rHuR proteins (150 ng loaded on gel) was not affected, while the phosphorylation of the protein was clearly induced, as showed by an anti-phosphoserine antibody (P-SER). **F**) Saturation binding experiments comparing the binding capability of rHuR and phosphorylated rHuR (P-rHuR). Nonlinear regression fits of the data revealed an equilibrium dissociation constants equal to 3.1±0.55 nM for P-rHuR, not statistically relevant (*P value* = 0.59) with respect to the *Kd* of rHuR. Mean and standard deviation values derive from two independent experiments.

In order to determine if this assay could be applied to evaluate competitive interactions we challenged the complex formation reaction by using untagged TNFα RNA probe (U-TNF). U-TNF was added to the reaction keeping constant the concentration of Bi-TNF (50 nM) and replicating the experimental condition of saturation binding. U-TNF would interfere with the thermodynamic equilibrium of the complex formation if a decrease of the signal intensity proportional to U-TNF amount is observed. Indeed, U-TNF nicely titrated the complex (*ki* of 4.49±1.1 nM) substituting Bi-TNF and increasing the percentage of inhibition in a dose dependent manner (Figure **3A**). Furthermore, we expressed the recombinant His-Strep-tagged TTP protein (rTTP) in HEK293T cells through strep-tactin resin purification (Figure **3B**). TTP competes with HuR intracellularly for binding to TNFα mRNA and this interplay dictates the translational efficiency of the target mRNA: HuR favors the polysomal recruitment of TNFα mRNA and facilitates its translation; in contrast, signaling cascades that activate TTP are responsible for the competition of this RBP against the same substrate leading to translational stop and/or RNA degradation [11,16]. We tested by REMSA the binding capability of purified rTTP by reacting a small amount of protein, as indicated, with 0.5 µM of Bi-TNF (Figure **3C**). In the gel, multiple protein-RNA probe complexes were observed, as also previously reported for TTP protein [21,22], demonstrating that rTTP was active and highly sensitive to this substrate. Competition AlphaScreen assays as function of rTTP concentration (0–3 nM) showed the direct interaction of rHuR and rTTP proteins towards the same Bi-TNF probe (Figure **3D**). According to our data, equilibrium dissociation constant *ki* of the competitive displacement was 1.76±0.41*10^-3^ nM, suggesting that rTTP has higher affinity than rHuR towards the ARE substrate and approximately ten-fold molar excess of rHuR seems necessary to initiate competition for displacement of rTTP. In this context, appropriate AlphaScreen assay should be designed to precisely measure rTTP binding kinetics and association/dissociation rate constants. To investigate how post-translational modifications, such as phosphorylations, could impact on the binding affinity of rHuR we stimulated transfected cells for 3 hr with cyclosporine (CsA) [4 µM], a compound able to induce in 786-0 renal cancer cells the HuR nucleo-cytoplasmic shuttling, its association with PKC-δ and its phosphorylation [23]. The CsA treatment in HEK293T cells did not affect the total amount of rHuR production in comparison with rHuR purified from control (DMSO) cells (Figure **3E**) and it clearly induced an heavy phosphorylation pattern on the recombinant protein (P-rHuR), detectable using an anti-phosphoserine antibody. Saturation binding experiments (Figure **3F**) revealed a not statistically significant *Kd* for P-rHuR (3.1±0.55 nM, *P value* = 0.59) and a not statistically significant association kinetics (data not shown), indicating that the phosphorylations induced by CsA do not modify the binding properties of the protein in this *in vitro* assay.

### AlphaScreen High Throughput Screening

**Figure 4:**
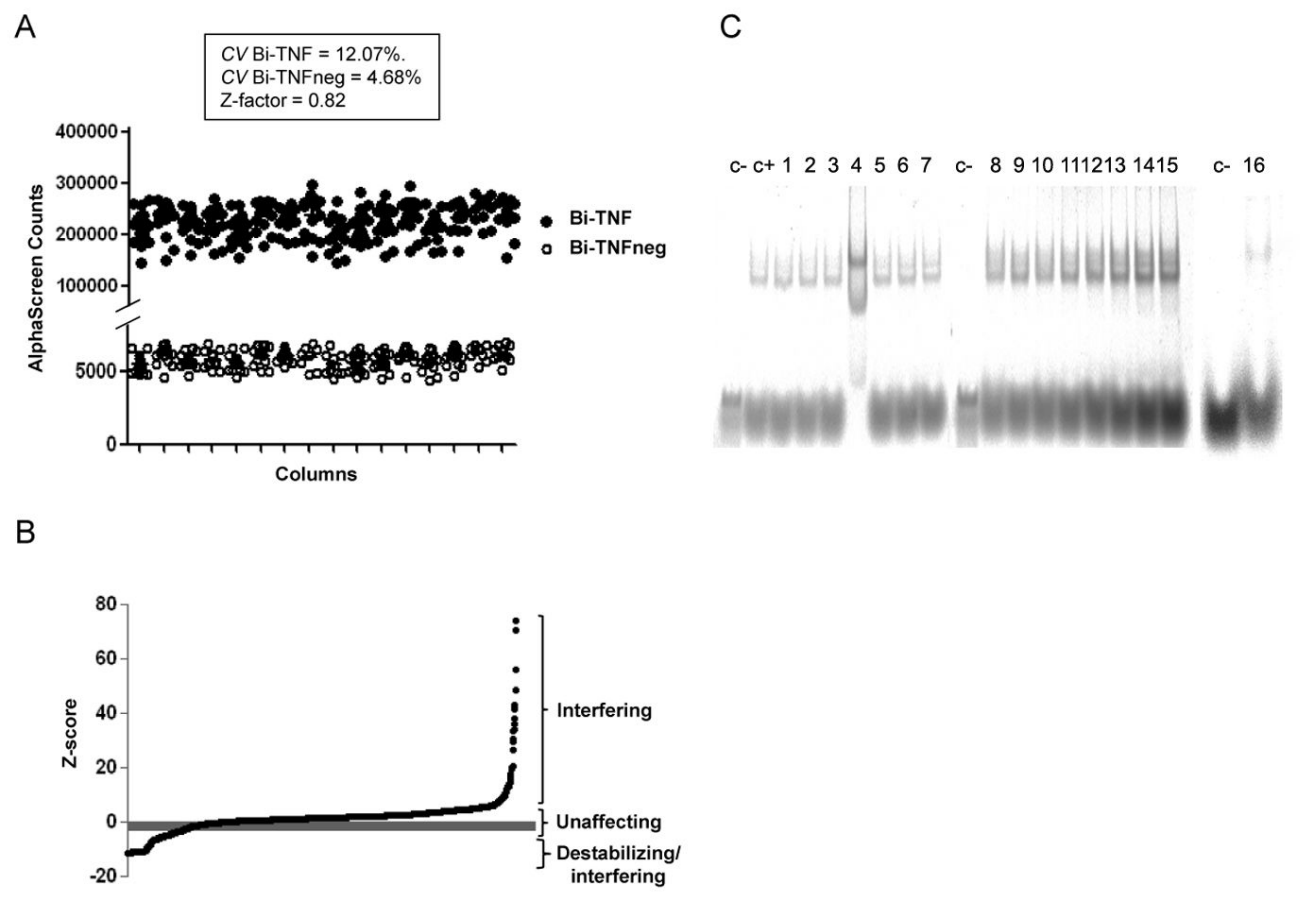
Robustness of the miniaturized AlphaScreen assay and screening of a drug library. **A**) rHuR and Bi-TNF positive or Bi-TNFneg negative controls were reacted at optimized nanomolar concentrations in a final volume of 20 µl in 384-wells Optiplates. Relative coefficient of variations and Z-factor value are reported. **B**) Plot of progressive Z-score values of 2000 compounds according to their interference to rHuR-RNA complex formation assay. **C**) Representative REMSA showing the effect of compounds, selected after counter screening assay, on rHuR-RNA complex formation. Lane 1: Bi-TNF probe only; Lane 2: rHuR-Bi-TNF; Lane 3-9: 1-Aspartame, 2-Cephradine, 3-Clomiphene citrate, 4-Cetylpyridinium chloride, 5-Diloxanide furoate, 6-Gentian violet, 7-Hydroquinone; Lane 10: Bi-TNF probe only; Lane 11-18: 8-Tilmicosin, 9-Nonoxynol-9, 10-Orlistat, 11-Protoveratrine, 12-Raloxifene hydrochloride, 13-Salsalate, 14-Switenolide diacetate, 15-Tetrandrine; Lane 19: Bi-TNF probe only; Lane 20: 16-Mitoxantrone hydrochloride. Compounds (0.5 µM) were added to a binding reaction containing, as in Line 2, 0.2 µM rHuR and 0.5 µM Bi-TNF.

As we added the same amount of rTTP and BSA after 20 min of rHuR-Bi-TNF incubation and the signals were detected 60 min later, we have thought that if rTTP had interfered with the thermodynamic equilibrium of the reaction we could exploit the AlphaScreen assay in a biochemical screening to identify molecules potentially targeting and inhibiting HuR-RNA interaction. In 384-well plate format we performed binding reaction with Bi-TNF positive and Bi-TNFneg negative controls. Coefficient of variations (CV) less than 15% and, importantly, a Z-factor value of 0.84 with a signal to background ratio (S/B) of 42 indicated that the assay was robust and reliable (Figure **4A**). As a pilot screening experiment, we tested a library of 2000 small molecules, at the final concentration of 25 nM, looking for candidate molecule able to inhibit the rHuR-Bi-TNF complex formation. Effectively, as shown in Figure **4B**, taking into account the induced auto-fluorescence of each compound and correction for unspecific binding, we ranked the library compounds according to their ability to affect the protein-RNA interaction (Full list is available in Table **S1**, Table **S2**). From the primary screening it has not been possible to discern between interfering compounds and real inhibitors of complex formation. Indeed interfering phenomena could be ascribed to technical artifacts, such as beads-molecule cross-talk or photochemical singlet oxygen generation/quenching, nevertheless to specific influence on RNA/rHuR conformational status. For these reason, 10% of hits classified as destabilizers in the primary screening were re-screened and the first 16 further tested by REMSA as secondary assay (Figure **4C**). Cethylpiridinium choride (lane 6) and Mitoxantrone (lane 16) clearly influenced the formation of the protein-RNA complex demonstrating the validity of the approach used and suggesting this method can be used in screening with wider chemical libraries.

## Discussion

We have recently applied the AlphaScreen technology to quantitatively discriminate the affinity of HuR towards RNA probes carrying ARE consensus sequences by comparing the binding to TNFα and p62 mRNA probes [24]. Here we finely dissect the potentiality of this assay measuring the binding kinetic properties, including association and dissociation rate constants, that regulate the binding of the full-length human recombinant HuR protein expressed in mammalian cells to the TNFα ARE-bearing mRNA probes. REMSA has been the tool of choice to investigate the binding capability of HuR towards target mRNAs since the first reported experiments [15]. However, although gel electrophoresis can give useful information about the molecular species present in the complex formation, REMSAs are cumbersome experiments, quantitative and qualitative amenable only for low throughput drug testing [25]. We focused our work to the development of a sensitive assay that could quantify the formation of the rHuR-RNA complex and that could be used in high throughput drug screening experiments. In this context, a previous approach has made use of recombinant truncated HuR protein and fluorescence intensity distribution analysis (FIDA) to specifically identify low-molecular-weight HuR inhibitors. By a mathematical model that could fit experimental evidences it has been shown HuR homodimerization before binding to RNA probe, with a stoichiometry of 2:1, protein: RNA respectively [20,26]. The strategy used in our assay allows to identify molecules that can interfere with complex formation, since the different competition of both U-TNF and rTTP specifically brought to a decrease on the ligands interactions. From saturation binding experiments we calculated a *Kd*, indistinguishable from the dissociation constant value of the *k*_*off*_ */k*_*on*_ ratio, quantified in the nanomolar range, indicating the sensitivity of the assay and the high affinity of the protein towards this RNA substrate. This addresses HuR-mRNA TNFα complex formation as a tightly controlled interaction as often highlighted in many reports [27–29]. Of note, observed association rate constants (data not shown) in time course experiments were linearly proportional to RNA probe concentration, describing a pseudo-first order association kinetic. However, precise determination of stoichiometric relationships between ligands is difficult here because estimation of Acceptor and Donor beads binding capacities and additional dissociation kinetic experiments are needed. Although we could not discriminate between the binding affinity of rHuR and P-rHuR to Bi-TNF we cannot exclude that further refinements of the assay such as site specific modifications of the post-translational pattern of the protein or a different design of the mRNA probe, would result in a differential binding affinity associated with post-translational modifications. Finally we report here the results of a 2000 molecules screening and the identification of a number of compounds that interfere with the assay or with rHuR-Bi-TNF complex formation. In the context of HTS applications, utilization of beads-based assays and, more importantly, of a readout that is strongly dependent on the redox species present in the binding buffer suggests particular care in the biological interpretation of data and, consequently, in their functional aspects. Consequently, hits are expected to be further characterized for mechanistic, pharmacological and biological relevance. Among the hits, Mitoxantrone revealed to be a compound interfering with rHuR binding to Bi-TNF in both of our *in vitro* assays. Since the anthracenedione scaffold was reported in previous screening for HuR inhibitors [26], we can consider the presence of this drug among our hits as a *bona fide* control of the robustness of our approach. This drug has been used for many years as an antitumoral agent [30], is a known ligand of nucleic acid [31] and it has been re-addressed for utilization in multiple sclerosis [32]. The inhibitory effect on complex formation we report here could be due to its unspecific property to precipitate mRNA [33], however mitoxantrone has been reported as a stabilizer of the tau pre-mRNA stem loop [34] after a screening of about 110000 molecules, suggesting the mRNA binding property of this molecule may relate to its pharmacology. Therefore it may also suggest that its inhibition activity of TNFα secretion [31] or its anticancer activity can partially depend on the interference with HuR-TNFα complex formation *in vivo*. Further studies are needed to elucidate this point. In summary, we have developed and characterized a quantitative and straightforward biochemical assay amenable for high throughput screening platforms. As a new tool it can open new perspectives in the elucidation of HuR binding ability in solution and may pave the way towards the identification of low-molecular-weight inhibitors specifically designed to break HuR-targeted mRNA interaction.

## Supporting Information

Figure S1 **Characterization of the functional binding of rHuR to the AU-Rich Element of TNFα and COX-2 3’ UTRs.** By saturation binding experiment, equilibrium dissociation constants (Kd) were determined from nonlinear regression fit of the data according to a 1-site binding model in GraphPad Prism®, version 5.0. *Kd* to Bi-COX is reported with standard error, *Kd* to Bi-TNF confirmed to be 2.751 nM.
(TIF)

Table S1 **List of 2000 molecules evaluated in the primary screening with corresponding Z-score**.
(XLS)

Table S2 **List of 218 molecules evaluated in the secondary screening with corresponding Z-score.** Molecules evaluated in REMSAs are the first 16 of the secondary screening.
(XLS)
