# Supplementary figures and images for: A Novel High Throughput Biochemical Assay to Evaluate the HuR Protein-RNA Complex Formation
Source: PLoS One. 2013 Aug 12;8(8):e72426. doi: 10.1371/journal.pone.0072426 (PMC3741180; doi:10.1371/journal.pone.0072426)

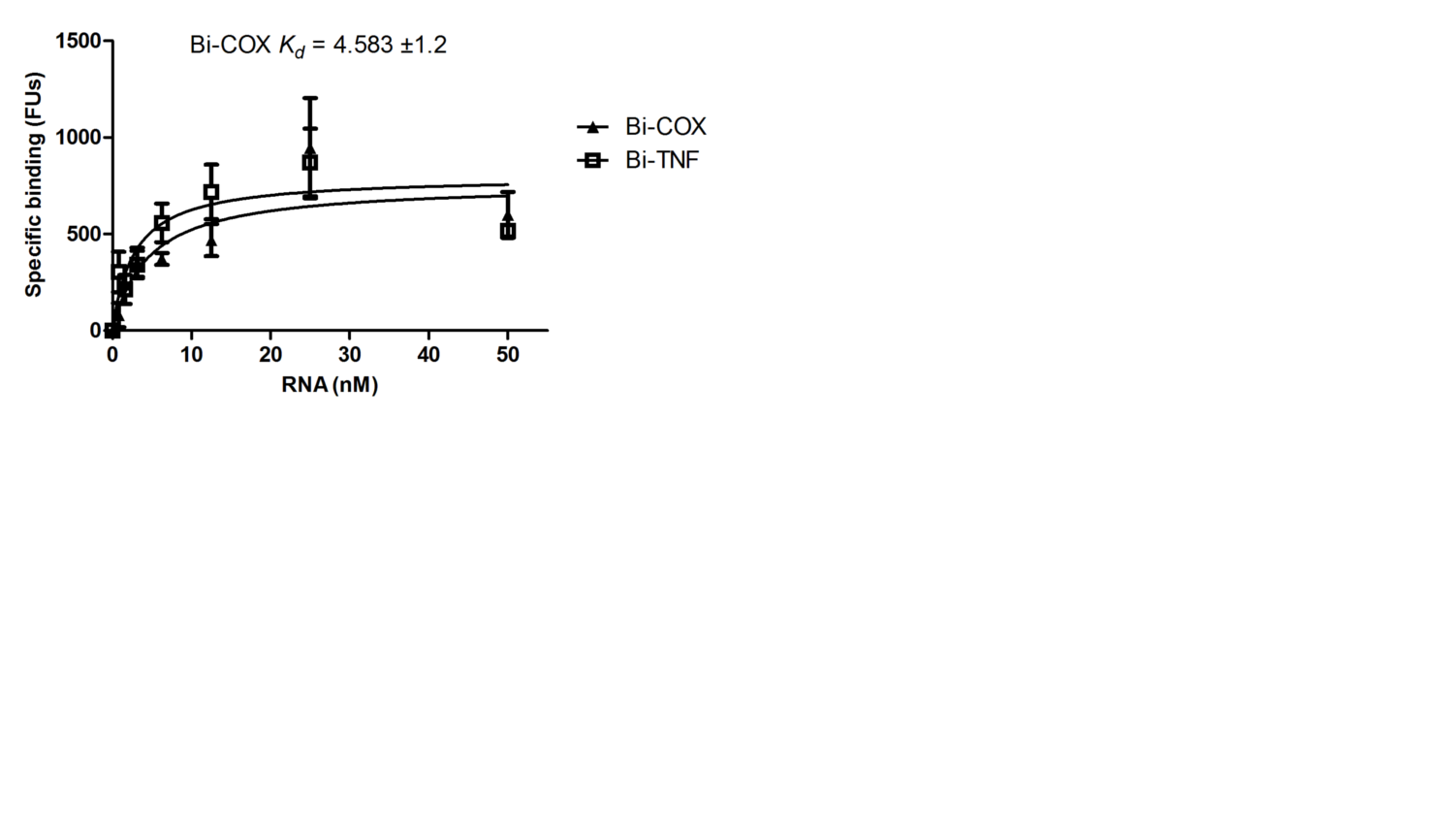

Supplement: Figure S1 — Characterization of the functional binding of rHuR to the AU-Rich Element of TNFα and COX-2 3’ UTRs. By saturation binding experiment, equilibrium dissociation constants (Kd) were determined from nonlinear regression fit of the data according to a 1-site binding model in GraphPad Prism®, version 5.0. Kd to Bi-COX is reported with standard error, Kd to Bi-TNF confirmed to be 2.751 nM. (TIF) [file pone.0072426.s001.tif]
